# Nicotine Oral Administration Attenuates DSS-Induced Colitis Through Upregulation of Indole in the Distal Colon and Rectum in Mice

**DOI:** 10.3389/fmed.2021.789037

**Published:** 2021-12-13

**Authors:** Akihito Nakajima, Tomoyoshi Shibuya, Takashi Sasaki, Yu Jie Lu, Dai Ishikawa, Keiichi Haga, Masahito Takahashi, Naoko Kaga, Taro Osada, Nobuhiro Sato, Akihito Nagahara

**Affiliations:** ^1^Department of Gastroenterology, Juntendo University, School of Medicine, Tokyo, Japan; ^2^Animal Research Center, Sapporo Medical University School of Medicine, Sapporo, Japan; ^3^Center of Excellence for Infection Control Science, Graduate School of Medicine, Juntendo University, Tokyo, Japan; ^4^Laboratory of Proteomics and Biomolecular Science, Research Support Center, Juntendo University Graduate School of Medicine, Tokyo, Japan

**Keywords:** nicotine, ulcerative colitis, indole, *Clostridium*, *Porphyromonas*, mice

## Abstract

Nicotine affects the gastrointestinal environment and modulates ulcerative colitis (UC). However, the associations among nicotine, gut metabolites, and UC are still largely unknown. We investigated whether orally administered nicotine affected gut metabolites and dextran sodium sulfate (DSS)-induced colitis. C57BL/6 male mice were orally administered nicotine solution in drinking water prior to inducing DSS-induced colitis. Short-chain fatty acids (SCFAs) and indole in gut contents and fecal samples were measured by GC-MS and hydroxylamine-based indole assays, respectively. Oral administration of nicotine increased indole concentration in feces, but, in contrast, SCFA values did not differ with nicotine administration. Indole levels were increased in the distal colon and rectum but not in the cecum and proximal colon. DSS-induced colitis was less severe clinically and histological changes were minimal in the rectum of orally nicotine-administered mice compared to mice drinking only water. 16S rRNA microbiome on the feces revealed an increasing in *Clostridium* and *Porphyromonas* in nicotine-administered mice. In conclusion, nicotine administration was associated with increased indole levels in the distal colon and rectum and attenuated DSS-induced colitis. Oral administration of nicotine may play a potential role in indole upregulation and prevention of UC.

## Introduction

That gut microbiota and its metabolites are involved in the immune response in the gut has been suggested (1, 2). Alterations of microbiota and metabolites are closely linked to many conditions including inflammatory bowel diseases (IBD) such as Crohn's disease (CD) and ulcerative colitis (UC). Dietary intake and cigarette smoking are major environmental factors that impact the gut microbiota and their metabolites (3–5). Cigarette smoking is considered a risk factor for many diseases such as lung cancer, heart diseases, and stroke. However, exceptionally, it has a protective effect against UC (6).

Nicotine is the main compound in cigarette products. Studies of nicotine and UC have suggested that nicotine has an anti-inflammatory effect in the gut (3, 7). However, why nicotine is protective against UC is still largely unknown, and the effect of nicotine on gut metabolites remains elusive.

Short-chain fatty acids (SCFAs), including acetate, propionate, and butyrate, are end-products of microbial fermentation and influence host physiology (8). Previous studies demonstrated that a soluble high fiber diet increased SCFA levels in the gut and attenuated DSS-induced colitis in mice (9, 10). That SCFAs provide energy to intestinal epithelial cells and affect the gut's immune system by inducing regulatory T (T_reg_) cells in the gut were shown (11, 12). In addition, we previously demonstrated that maternal SCFAs promoted the development of T_reg_ cells in offspring (13, 14). As SCFAs play many crucial roles in the immune system, it is important to study whether nicotine is involved in SCFA production and protects against colitis.

Indole is a gut bacterial metabolite derived from tryptophan (Trp). Indole is the most abundant Trp metabolite and plays a crucial role in the intestinal immune system (15, 16). Various levels of Trp metabolites are associated with UC disease activity (17). Additionally, indole compounds have been used for the treatment for UC (18). Although indole plays a pivotal role in the gut, it is not clear whether nicotine influences indole production in the gut. In this study, we aimed to investigate whether the oral administration of nicotine influenced gut metabolites such as SCFAs and indole and also examined if such administration had an influence on dextran sodium sulfate (DSS)-induced colitis.

## Results

### Oral Administration of Nicotine Increased Fecal Indole Concentration and Attenuated DSS-Induced Colitis

**Figure 1:**
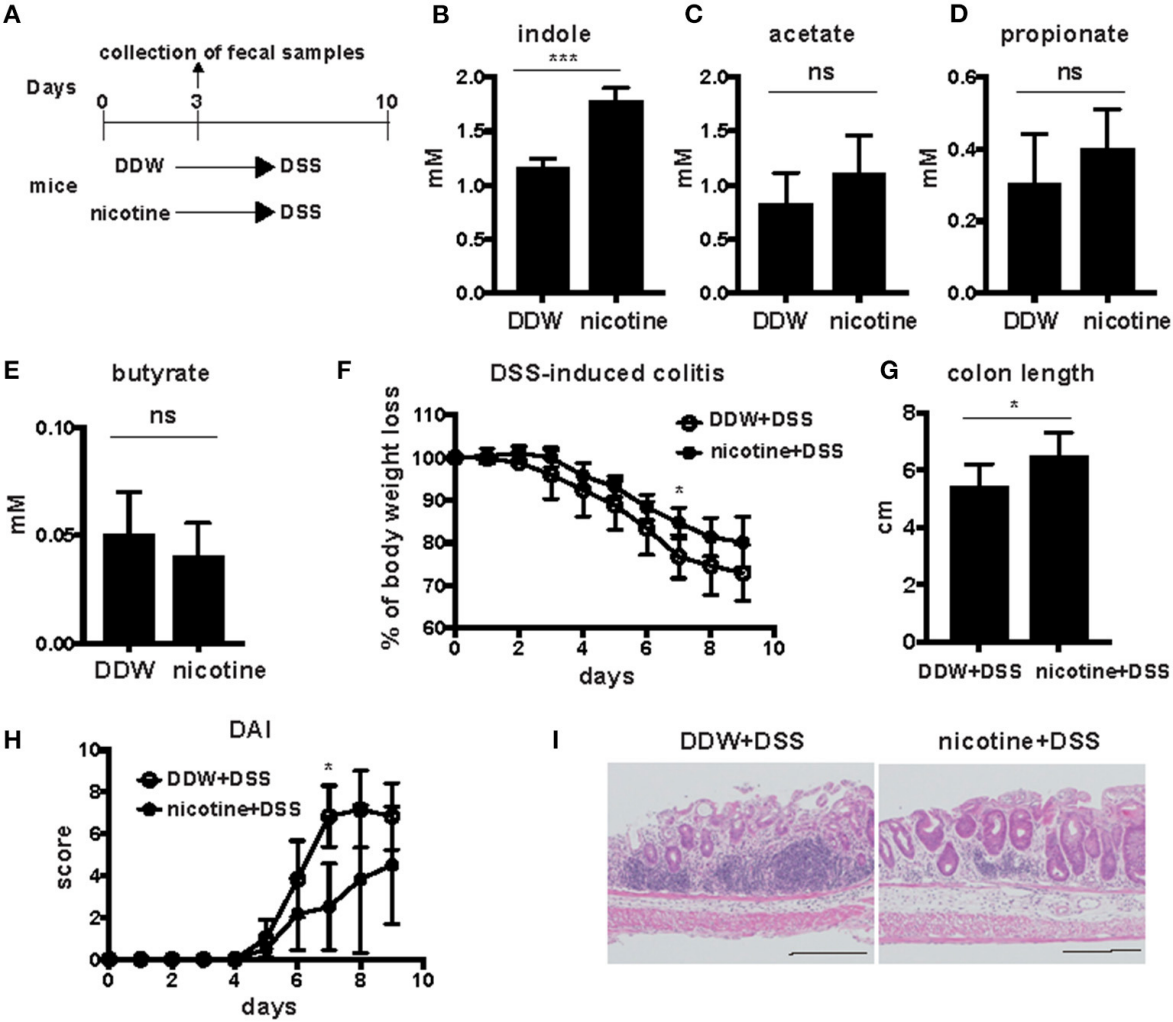
Twenty microgram/milliliter of orally administered nicotine increased indole concentration and attenuated DSS-induced colitis. **(A)** Schema of the experiments in this figure. DDW or 20 μg/ml of nicotine dissolved in drinking water was orally administered for 3 days. Fecal samples were collected at day 3. Then, 2.5% DSS was orally administered in drinking water for 7 days to mice drinking only DDW and nicotine-administered mice. DSS drinking water was switched to DDW for the next 2 days. **(B–E)** Indole levels in feces from mice drinking only DDW and nicotine-administered mice were measured by the HIA method. *N* = 5 mice per group. Data are shown as mean ± SD. ***P < 0.001. Acetate **(C)**, propionate **(D)**, and butyrate **(E)** in feces of mice drinking only DDW and nicotine-administered mice were measured by GC-MS. Concentrations of SCFAs were the average of two runs. *N* = 7 mice per group. ns; not significant. **(F–I)** Body weight **(F)** was measured every day and colon length **(G)** was measured at Day 9. *N* = 5 mice per group. Data are presented as mean ± SD. *P < 0.05. **(H)** The DAIs of mice drinking only DDW and nicotine-administered mice were scored in total for the following categories: weight loss, 0, no loss; 1, 5–10% loss; 2, 10–15% loss; 3, 15–20% loss; and 4, 20% weight loss; for stool consistency,: 0, normal; 1, mild loose stool; 2, moderate loose stool; and 3, diarrhea; for bleeding, 0, no blood; 1, presence of blood; and 2, gross blood. *N* = 5 mice per group. Data are presented as mean ± SD. *P < 0.05. **(I)** Histological analysis of representative sections of the rectum with H&E staining from mice drinking only DDW and nicotine-administered mice after DSS-induced colitis. Black bars in lower right side indicated 200 μm.

To address the effect of nicotine on gut metabolites, wild-type (WT) mice were orally administered 20 μg/ml of nicotine solution dissolved in drinking water for 3 days, after which levels of indole and SCFAs in feces were analyzed. Previous studies demonstrated that low doses of oral nicotine prevented DSS-induced colitis (19, 20). Indole concentrations in feces were detected by the Hydroxylamine-based indole assay (HIA) method (21). The mean level of indole in feces of WT mice that were provided only deionized distilled water (DDW) was 1.16 mM and that in nicotine-administered mice was 1.77 mM (Figure 1B), suggesting that oral administration of nicotine significantly increased the fecal indole level. In contrast, fecal levels of SCFAs, such as acetate, propionate, and butyrate, did not differ between mice drinking only DDW and nicotine-administered mice (Figures 1C–E). This result suggested that oral administration of nicotine affected fecal levels of indole but not SCFAs. DSS-induced colitis is considered to be a mouse model of UC; thus, DSS was orally administered for 7 days to mice drinking only DDW and nicotine-administered mice subsequent to the oral administration of nicotine to examine the effect of nicotine on this mouse model of UC (Figure 1A). DSS-induced colitis in mice orally administered nicotine significantly decreased the percentage of body weight loss on Day 7 (Figure 1F). The mean intestinal length of the DDW group was significantly shorter than the mean intestinal length of the nicotine group (Figure 1G). Furthermore, clinical signs such as weight loss, stool consistency, and bleeding were scored as the disease activity index (DAI). DAI scores in nicotine-administered mice were significantly lower than in mice drinking only water at Day 7 (Figure 1H). Histological analysis of the rectum also showed that infiltration of lymphocytes and the damage to crypts in nicotine-administered mice were less than in mice drinking only DDW (Figure 1I).

Those results suggested that oral administration of nicotine attenuated DSS-induced colitis and that increased indole values might be associated with the protective effect of nicotine administration on DSS-induced colitis.

### Levels of Indole Were Increased in the Distal Colon and Rectum

**Figure 2:**
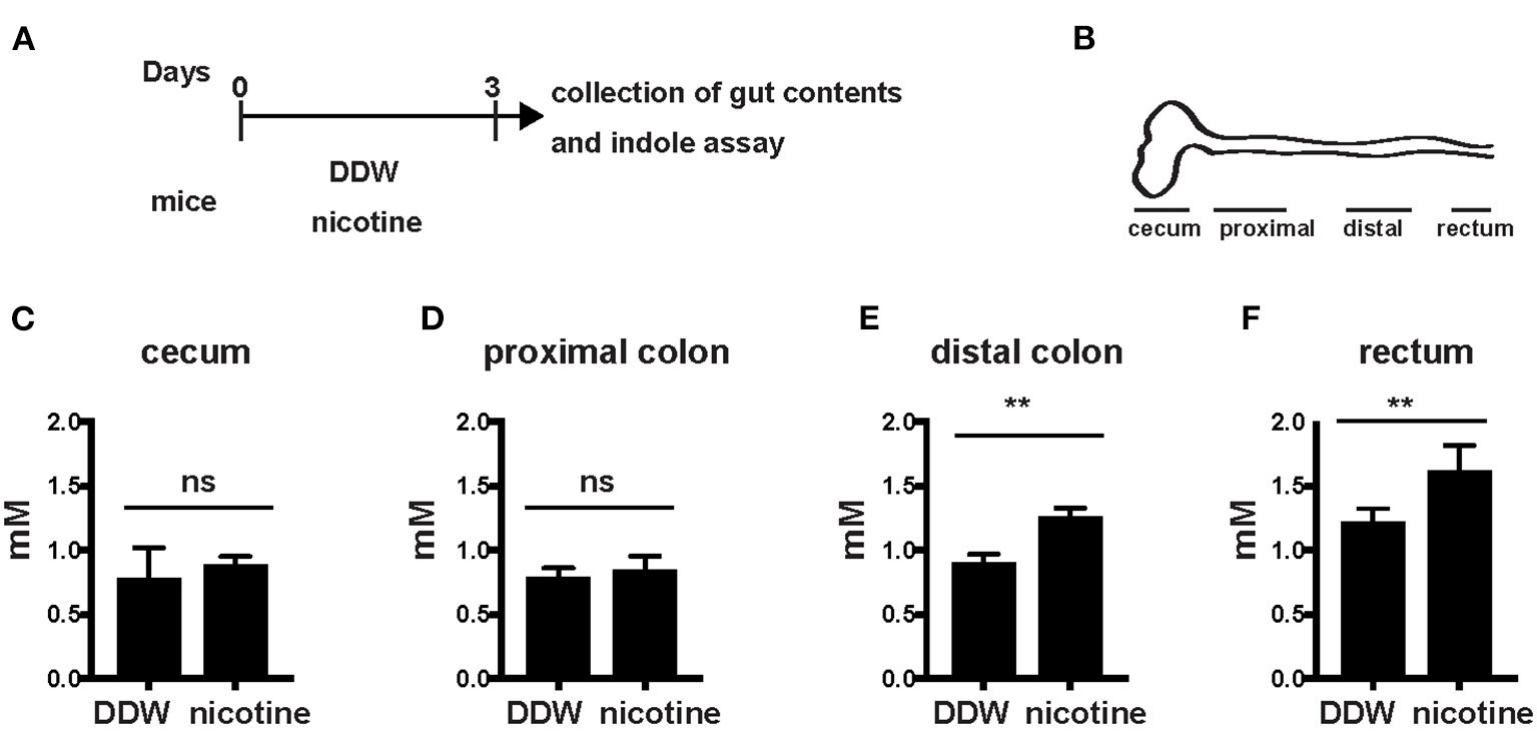
Indole levels in the distal colon and rectum were increased in nicotine- administered mice. **(A)** Schema of the experiments in this figure. DDW or 20 μg/ml of nicotine was orally administered in drinking water for 3 days. Then, mice were sacrificed and the gut contents from the cecum, proximal colon, distal colon, and rectum were collected at day 3. **(B)** Schema of the colon divided into the cecum, proximal colon, distal colon, and rectum. **(C–F)** Gut contents of the cecum, proximal colon, distal colon, and rectum from mice drinking only DDW and nicotine-administered mice were collected and indole concentrations in the cecum **(B)**, proximal colon **(C)**, distal colon **(D)**, and rectum **(E)** were measured by the HIA method. *N* = 5 mice, per group. Data are shown as mean ± SD. **P < 0.01. ns, not significant.

Next, we further analyzed levels of indole in the gut according to oral administration of nicotine. WT mice were orally administered DDW or nicotine solution dissolved in drinking water to nicotine-administered mice for 3 days (Figure 2A). Indole concentration in the cecum and the entire colon divided into the proximal colon, distal colon, and rectum was analyzed in both mice drinking only DDW and nicotine-administered mice (Figure 2B). Indole levels in the cecum and proximal colon did not differ between mice drinking only DDW and nicotine-administered mice (Figures 2C,D) but, interestingly, indole levels in the distal colon and rectum were significantly increased in nicotine-administered mice (Figures 2E,F). In the mice drinking only DDW group, mean indole concentrations in the cecum, proximal colon, distal colon, rectum, and feces varied from 0.77, 0.68, 0.90, 1.22, and 1.16 mM, respectively (Figures 1B, 2C–F). Indole concentration in the rectum was almost twice as high as in the proximal colon in mice drinking only DDW. In the rectum, mean indole concentrations in mice drinking only DDW and nicotine-administered mice were 1.21 and 1.61 mM, respectively (Figure 2F). Thus, indole concentration was 1.5-fold higher in the rectum in the nicotine-administered mice than in mice drinking only DDW.

### The Abundance of Clostridium and Porphyromonas Were Significantly Increased in the Nicotine-Administered Mice

**Figure 3:**
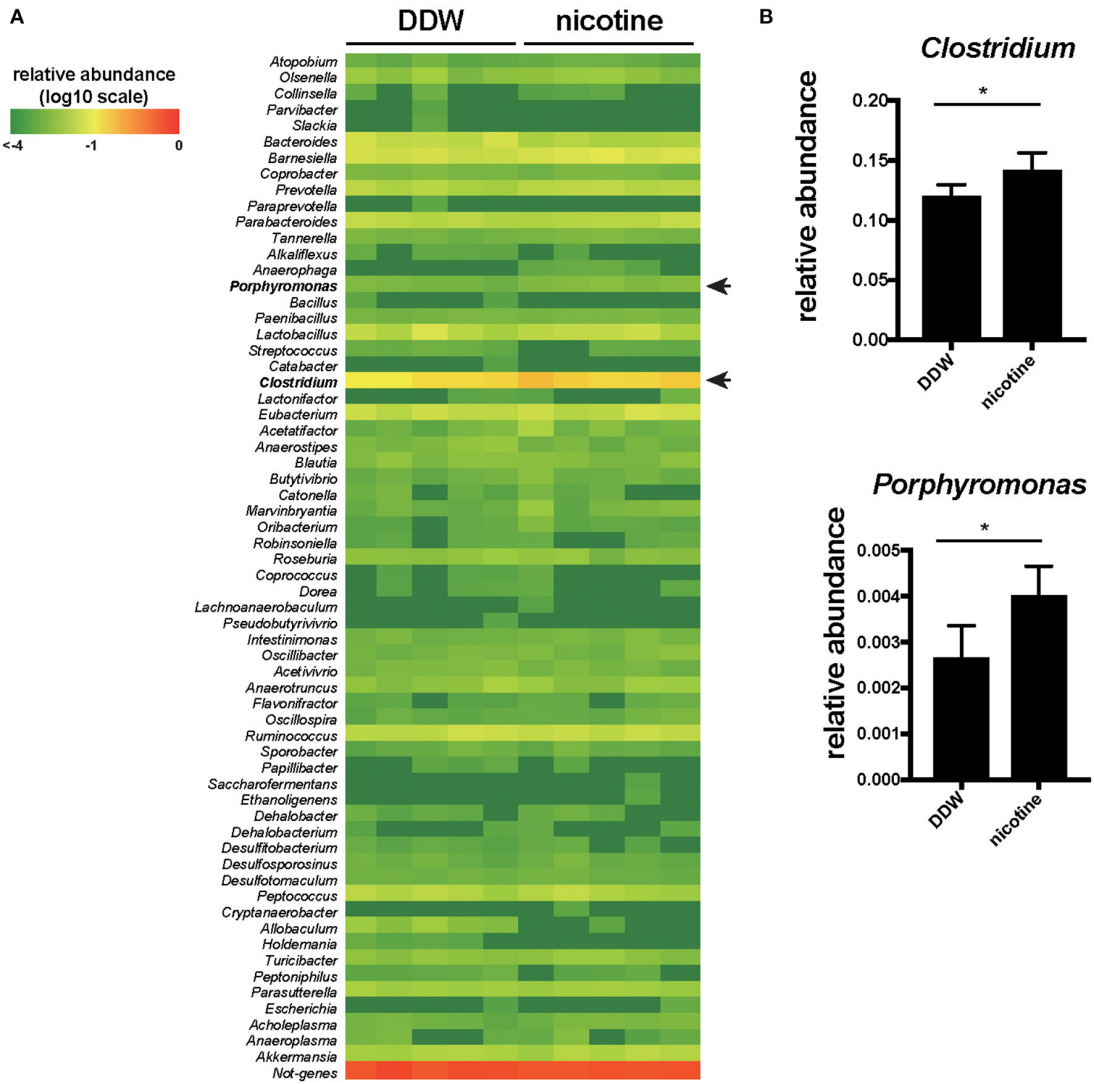
The abundance of *Clostridium* and *Porphyromonas* were significantly increased in the nicotine-administered mice. **(A)** The fecal samples from mice drinking only DDW and nicotine-administered mice were analyzed for 16S rRNA microbiome at the genus level. The heatmap showing the relative abundances of 63 genus in the feces. The relative value for each bacterial genus is indicated by color intensity (legend indicated at the top left corner). *N* = 5 mice per group. **(B)** The relative abundance of *Clostridium* and *Porphyromonas* of DDW and nicotine-administered mice. *N* = 5 mice per group. Data are presented as mean ± SD. *P < 0.05, a Wilcoxon rank sum test.

Then, we analyzed the composition of the gut microbiota of feces by 16S rRNA microbiome method to reveal the association of nicotine, indole and the gut microbiota in mice drinking only DDW and nicotine-administered mice (Figure 3A). *Clostridium* and *Porphyromonas* were significantly increased (*p* < 0.05, a Wilcoxon rank sum test) in the nicotine-administered mice compared to DDW mice (Figure 3B).

Overall, production of indole was higher in the distal colon and rectum than in the cecum and proximal colon. Thus, oral administration of nicotine appeared to promote indole production in the distal colon and rectum. The indole level in the rectum and feces was associated with the severity of DSS-induced colitis.

## Discussion

In this study, we revealed an association of indole with the severity of DSS-induced colitis in mice orally administered nicotine. We detected major metabolites, SCFAs, and indole in gut contents and feces of orally administered nicotine mice and showed that indole levels in the rectum and feces were increased in nicotine-administered mice and that the severity of DSS-induced colitis was attenuated. Indole is the most abundant bacterial-derived Trp catabolite. It has been reported that low levels of Trp metabolites are a risk factor for and are associated with inflammatory bowel diseases (IBD) (17). In fact, serum levels of Trp were lower in IBD patients and Trp-deficient mice manifested severe colitis (17, 22). Trp catabolites, including indole and indole derivatives, mediate microbial signals (23). Indole contributed to epithelial barrier function by increasing certain molecules involved in tight junctions and adherence junctions (15, 16). It was reported that indole concentration was decreased in germ-free (GF) mice; however indole treatment enhanced associated molecules of tight junctions and adherence junctions, and attenuated DSS-induced colitis in GF mice (15). This indicated that upregulation of indole resulted in strengthening of epithelial barrier function and tight junctions in mice. In line with this result, we demonstrated here that upregulated indole in the distal colon and rectum through oral administration of nicotine strengthened barrier function. We observed attenuated DSS-induced colitis in the nicotine-administered mice (Figures 1F–H). Histological analysis revealed that colonic damage, such as loss of crypts and goblet cells and infiltration of inflammatory cells, was less severe in the rectum of such mice (Figure 1I). In the rectum, increased indole attenuated DSS-induced colitis, suggesting that increased indole by nicotine administration attenuated UC through epithelial barrier function. As previously reported, oral administration of 40 kDa DSS solution, such as used in this study, was associated with more severe colitis in the distal colon and rectum than in the proximal colon (24). Upregulation of indole concentration in the distal colon and rectum may have a protective effect against colorectal damage by DSS-induced colitis.

Trp metabolites including indole and indole derivatives act as ligands of aryl hydrocarbon receptor (AhR). AhR is a transcriptional factor that regulate host immune system. For example, activation of AhR enhances Interleukin-22 production in type 3 innate lymphoid cells, which contributes to epithelial barrier function (25, 26). Based on these studies, AhR pathway may be involved in the attenuation of UC by nicotine administration.

The gut microbiota is closely linked to the gut metabolites. In this study, we showed increased the relative abundance of *Clostridium* and *Porphyromonas* by 16S rRNA microbiome method on fecal samples in the nicotine-administered mice. Previous studies have also demonstrated that cigarette smoking and nicotine treatment affect the composition of the gut microbiota in human and mice (4, 27–30). The relative abundance of *Bacteroides*-*Prevotella* and the Clostridia family *Veillonellaceae* were higher in current smokers (28, 31). In a mice model, it has been reported that the relative abundance of *Clostridium clostridiforme* was increased (29). According to the previous reports, indole is produced from Trp by tryptophanase encoded *tnaA* gene and some *Clostridium* and *Porphyromonas* species contain the *tnaA* gene and produce indole (23, 32). Increased *Clostridium* and *Porphyromonas* in nicotine-administered mice may contribute to promote the level of indole. Some species of *Bacteroides* and *Prevotella*, as well as *Clostridium* and *Porphyromonus* contained *tnaA* gene are thought to be involved in producing indole (23, 32).

Cigarette smoking is a major risk factor for Crohn's disease; however it is protective against the development and progression of UC (6, 33). UC is a chronic mucosal inflammation in the gut and is sub-classified into the following three types: ulcerative proctitis, left-sided UC, and extensive UC (34). Colonic mucosal inflammation of UC starts in the rectum and extends proximally in a continuous fashion (35). Therefore, to protect against rectal inflammation is a crucial strategy for UC treatment. Notably, we demonstrated here that nicotine affected indole concentrations in the distal colon and rectum rather than in the proximal colon. That orally administered nicotine increased indole in the rectum and attenuated DSS-induced colitis raised the therapeutic possibility of this strategy for UC patients although there might be a limitation to generalize the effect of nicotine.

In conclusion, we propose that increased indole by oral administration of nicotine has a potential role to protect the rectum, which is an important site for UC progression. Indole is a key metabolite and nicotine might have functions to protect against UC through upregulation of indole. Further studies should be required to clarify the mechanism by which nicotine increased indole.

## Materials and Methods

### Mice

C57BL/6J male mice at 10 weeks old were purchased from Japan SLC (Shizuoka, Japan). Mice were housed at the animal facility of Juntendo University (Tokyo, Japan) and fed a normal diet, CRF-1 (Orietal Yeast, Tokyo, Japan). Five or seven mice were housed in each gauge under standard 12 h light/dark cycles, 22 ± 2 degrees, and 50 ± 5 percent humidity. All animal experiments were approved by the Animal Experimentation Committee of Juntendo University (No. 2021122).

### Reagents

(–)-Nicotine hydrogen tartrate salt was purchased from Sigma (St. Louis, MO, USA). Dextran sodium sulfate (M.W 36000-50000) was purchased from MP Biomedicals (Santa Ana, CA, USA).

### Hydroxylamine-Based Indole Assay (HIA)

HIA was performed according to a previous paper describing the determination of indole concentration (21). Briefly, gut content samples were diluted with 70% ethanol to 100 mg/ml and disrupted by a Power Masher II (Nippi, Tokyo, Japan). After centrifugation, supernatants were filtered using a Millipore Ultrafree MC PLHCC centrifugal filter (Merck Millipore, Billerica, MA, USA). Samples were incubated for 15 min at room temperature with 5M NaOH (Wako, Osaka, Japan) and 50 μl of 0.3 M hydroxylamine hydrochloride (Wako) in a microtiter plate. Following incubation, 125 μl of 2.5 M H_2_SO_4_ (Wako) was added and incubated at room temperature for 30 min. The plates were immediately read at 530 nm using optical density readings by a microplate reader.

### Measurement of SCFA Concentration in Feces

Hundred milligrams of fresh feces from C57BL/6 male mice were homogenized in 400 μl H_2_O containing hexanoic acid (methyl-d_3_) as an internal standard. Then 80 μl of 25% meta-phosphoric acid was added to the homogenate and kept on ice for 30 min. Thereafter, samples were centrifuged at 17,500 × *g* for 15 min at 4°C. The supernatants were filtered using a Millipore Ultrafree MC PLHCC centrifugal filter (Merck Millipore) and analyzed by gas chromatography-mass spectrometry (GC-MS). One microliter of the sample was injected with a split mode (1:100) into a TRACE GC ULTRA gas chromatograph equipped with a TSQ QUANTUM GC mass spectrometer (ThermoFisher Scientific, Waltham, MA). A Nukol™ fused silica capillary column (0.25 mm ID × 30 m, 0.25 μm film thickness; Supelco, Bellefonte, PA, USA) was used for separation. Column temperature was programmed for 150°C for 2 min, then increased to 200°C at a rate of 8°C/min and held at 200°C for 13 min. Helium was used as a carrier gas at a flow rate of 0.7 ml/min. Data were acquired by the electron impact ionization mode at 70 eV.

### Dextran Sulfate Sodium (DSS)-Induced Colitis

C57BL/6J male mice at 10 weeks old were orally administered nicotine [(–)-Nicotine hydrogen tartrate salt, Sigma] in drinking water at 20 μg/ml for 3 days. Then, 2.5% DSS (M.W 36,000–50,000; MP Biomedicals) dissolved in sterile, distilled drinking water was orally administered for 7 days. On day 7, 2.5% was switched to regular drinking water for the next 2 days. Body weight was measured every day. On day 9, mice were sacrificed and colon lengths were measured.

### Microbiome Analysis Based on 16S rRNA Amplicon Sequencing

The fecal samples 3 days after DDW and nicotine administration were diluted 10-fold in TE buffer were diluted 10-fold in TE buffer (10 mM Tris, 1 mM EDTA [pH 8.0]) and frozen at −80°C until use. Five hundred microliter of each diluted sample was used for DNA extraction. After pretreatment in TE buffer with 50 U of achromopeptidase (Wako) at 50°C for 30 min, phenol: chloroform: isoamyl alcohol (25:24:1, v/v/v) was used for DNA purification.

We performed an Illumina 16S metagenomic sequencing protocol, which targeted the V3 to V4 region of bacterial and archaeal 16S rRNA genes, for comprehensive analysis of the fecal microbiota, following the manufacturer's workflow of 16S Metagenomic Sequencing Library Preparation, recommended by Illumina. We used 16S universal primers without adapter sequences, 16S amplicon PCR forward primer (5′-TCGTCGGCAGCGTCAGATGTGTATAAGAGACAGCCTACGGGNGGCWGCAG-3′) and 16S amplicon PCR reverse primer (5′-GTCTCGTGGGCTCGGAGATGTGTATAAGAGACAGGACTACHVGGGTATCTAATCC-3′). The PCR reaction mixture consisted of two μL of DNA extract in a total volume of 25 μL containing 1× KAPA HiFi HotStart ReadyMix (KAPA Biosystems, Boston, MA, USA) and 10 pmol of each primer. Reaction mixtures were thermally cycled once at 95°C for 2 min; then 25–30 times at 95°C for 30 s, 65°C for 30 s, and 72°C for 90 s; and then once at 72°C for 2 min. DNA fragments were analyzed by electrophoresis in TAE buffer on a 1% agarose gel stained with ethidium bromide. The PCR products were then purified using AMPure beads (Beckman Coulter, Inc., CA), according to the manufacturer's protocol. PCR products were uniquely indexed using a Nextera XT Index Kit (Illumina, San Diego, CA, USA). NucleoMag NGS Cleanup and Size Select technology (Macherey-Nagel, Düren, Germany) was used twice for cleanup and size selection of NGS libraries, according to a protocol for removing adapter dimers. Sequencing was performed using a Miseq reagent kit v3 (600 Cycle) and a paired-end 2× 300-bp cycle run on an Illumina MiSeq sequencing system (Illumina, San Diego, CA, USA).

MiSeq-read 1 and 2 reads were stitched by FLASH (36). The merged reads were filtered and trimmed by removing bases with quality value (QV) scores of 20 or less and read lengths shorter than 200 bases, and then converted from FASTQ to FASTA format using FASTX toolkit ver. 0.0.14 (http://hannonlab.cshl.edu/fastx_toolkit). Analyses of the trimmed sequencing reads were performed using blastn by blast 2.5.0+, with an e^−10^ e-value cutoff (37). Taxonomic classification was performed using MEGAN version 5 (38).

### Histological Analysis

Entire colons were cut longitudinally and fixed in 10% formalin and embedded in paraffin. 3-μm sections of the rectum were stained with hematoxylin and eosin (H&E).

### Statistical Analysis

Statistical analysis was performed using the Student's *t* test. GraphPad Prism 7 (GraphPad Software, La Jolla, CA, USA) was used for all statistical calculations. We performed a Wilcoxon rank sum test against bacterial genus exhibiting increase or decrease in relative abundance. A *p*-value of <0.05 was considered statistically significant.

## Data Availability Statement

The original contributions presented in the study are publicly available. This data can be found here: https://www.ddbj.nig.ac.jp/, DRA012847.

## Ethics Statement

The animal study was reviewed and approved by the Animal Experimentation Committee of Juntendo University (No. 2021122).

## Author Contributions

ANak and TSh conceived this project. ANak designed the study and performed most experiments. TSa and YL performed 16S rRNA microbiome and analysis of the data. NK performed measurement of SCFAs concentration in feces. TSh, TO, NS, and ANag supervised the study. ANak and TSh wrote the manuscript. All authors interpreted the data and approved the final version of the manuscript.

## Funding

This study received funding from Takeda Research Support (No. TKDS20180424018) and research grant from Mitsubishi Tanabe Pharma Corporation (No. MTPS20200527020 and MTPS20190518004). The funders were not involved in the study design, collection, analysis, interpretation of data, the writing of this article or the decision to submit it for publication.

## Conflict of Interest

The authors declare that the research was conducted in the absence of any commercial or financial relationships that could be construed as a potential conflict of interest.

## Publisher's Note

All claims expressed in this article are solely those of the authors and do not necessarily represent those of their affiliated organizations, or those of the publisher, the editors and the reviewers. Any product that may be evaluated in this article, or claim that may be made by its manufacturer, is not guaranteed or endorsed by the publisher.

## References

[1] Lavelle A Sokol H. Gut microbiota-derived metabolites as key actors in inflammatory bowel disease. Nat Rev Gastroenterol Hepatol. (2020) 17:223–7. 10.1038/s41575-019-0258-z 32076145

[2] Rooks MG Garrett WS. Gut microbiota, metabolites and host immunity. Nat Rev Immunol. (2016) 16:341–52. 10.1038/nri.2016.42 27231050 PMC5541232

[3] Berkowitz L Schultz BM Salazar GA Pardo-Roa C Sebastian VP Alvarez-Lobos MM. Impact of cigarette smoking on the gastrointestinal tract inflammation: opposing effects in Crohn's Disease and Ulcerative Colitis. Front Immunol. (2018) 9:74. 10.3389/fimmu.2018.00074 29441064 PMC5797634

[4] Lee SH Yun Y Kim SJ Lee EJ Chang Y Ryu S. Association between cigarette smoking status and composition of gut microbiota: population-based cross-sectional study. J Clin Med. (2018) 7:282. 10.3390/jcm7090282 30223529 PMC6162563

[5] Savin Z Kivity S Yonath H Yehuda S. Smoking and the intestinal microbiome. Arch Microbiol. (2018) 200:677–84. 10.1007/s00203-018-1506-2 29626219

[6] Parkes GC Whelan K Lindsay JO. Smoking in inflammatory bowel disease: impact on disease course and insights into the aetiology of its effect. J Crohns Colitis. (2014) 8:717–25. 10.1016/j.crohns.2014.02.002 24636140

[7] Lakhan SE Kirchgessner A. Anti-inflammatory effects of nicotine in obesity and ulcerative colitis. J Transl Med. (2011) 9:129. 10.1186/1479-5876-9-129 21810260 PMC3163205

[8] den Besten G van Eunen K Groen AK Venema K Reijngoud DJ Bakker BM. The role of short-chain fatty acids in the interplay between diet, gut microbiota, and host energy metabolism. J Lipid Res. (2013) 54:2325–40. 10.1194/jlr.R036012 23821742 PMC3735932

[9] Nakajima A Sasaki T Itoh K Kitahara T Takema Y Hiramatsu K. A Soluble fiber diet increases bacteroides fragilis group abundance and immunoglobulin a production in the gut. Appl Environ Microbiol. (2020) 86:e00405–20. 10.1128/AEM.00405-20 32332136 PMC7301863

[10] Makki K Deehan EC Walter J Backhed F. The impact of dietary fiber on gut microbiota in host health and disease. Cell Host Microbe. (2018) 23:705–15. 10.1016/j.chom.2018.05.012 29902436

[11] Furusawa Y Obata Y Fukuda S Endo TA Nakato G Takahashi D. Commensal microbe-derived butyrate induces the differentiation of colonic regulatory T cells [Research Support, Non-U.S. Gov't]. Nature. (2013) 504:446–50. 10.1038/nature12721 24226770

[12] Arpaia N Campbell C Fan X Dikiy S van der Veeken J deRoos P. Metabolites produced by commensal bacteria promote peripheral regulatory T-cell generation. Nature. (2013) 504:451–5. 10.1038/nature12726 24226773 PMC3869884

[13] Nakajima A Kaga N Nakanishi Y Ohno H Miyamoto J Kimura I. Maternal high fiber diet during pregnancy and lactation influences regulatory T cell differentiation in offspring in mice. J Immunol. (2017) 199:3516–24. 10.4049/jimmunol.1700248 29021375

[14] Nakajima A Habu S Kasai M Okumura K Ishikawa D Shibuya T. Impact of maternal dietary gut microbial metabolites on an offspring's systemic immune response in mouse models. Biosci Microbiota Food Health. (2020) 39:33–8. 10.12938/bmfh.19-013 32328398 PMC7162694

[15] Shimada Y Kinoshita M Harada K Mizutani M Masahata K Kayama H. Commensal bacteria-dependent indole production enhances epithelial barrier function in the colon. PLoS One. (2013) 8:e80604. 10.1371/journal.pone.0080604 24278294 PMC3835565

[16] Bansal T Alaniz RC Wood TK Jayaraman A. The bacterial signal indole increases epithelial-cell tight-junction resistance and attenuates indicators of inflammation. Proc Natl Acad Sci U S A. (2010) 107:228–33. 10.1073/pnas.0906112107 19966295 PMC2806735

[17] Nikolaus S Schulte B Al-Massad N Thieme F Schulte DM Bethge J. Increased tryptophan metabolism is associated with activity of inflammatory bowel diseases. Gastroenterology. (2017) 153:1504–1516 e2. 10.1053/j.gastro.2017.08.028 28827067

[18] Sugimoto S Naganuma M Kanai T. Indole compounds may be promising medicines for ulcerative colitis. J Gastroenterol. (2016) 51:853–61. 10.1007/s00535-016-1220-2 27160749

[19] AlSharari SD Akbarali HI Abdullah RA Shahab O Auttachoat W Ferreira GA. Novel insights on the effect of nicotine in a murine colitis model. J Pharmacol Exp Ther. (2013) 344:207–17. 10.1124/jpet.112.198796 23115221 PMC3533410

[20] Eliakim R Karmeli F Cohen P Heyman SN Rachmilewitz D. Dual effect of chronic nicotine administration: augmentation of jejunitis and amelioration of colitis induced by iodoacetamide in rats. Int J Colorectal Dis. (2001) 16:14–21. 10.1007/s003840000262 11317692

[21] Darkoh C Chappell C Gonzales C Okhuysen P. A rapid and specific method for the detection of indole in complex biological samples. Appl Environ Microbiol. (2015) 81:8093–7. 10.1128/AEM.02787-15 26386049 PMC4651089

[22] Hashimoto T Perlot T Rehman A Trichereau J Ishiguro H Paolino M. ACE2 links amino acid malnutrition to microbial ecology and intestinal inflammation. Nature. (2012) 487:477–81. 10.1038/nature11228 22837003 PMC7095315

[23] Roager HM Licht TR. Microbial tryptophan catabolites in health and disease. Nat Commun. (2018) 9:3294. 10.1038/s41467-018-05470-4 30120222 PMC6098093

[24] Kitajima S Takuma S Morimoto M. Histological analysis of murine colitis induced by dextran sulfate sodium of different molecular weights. Exp Anim. (2000) 49:9–15. 10.1538/expanim.49.9 10803356

[25] Zelante T Iannitti RG Cunha C De Luca A Giovannini G Pieraccini G. Tryptophan catabolites from microbiota engage aryl hydrocarbon receptor and balance mucosal reactivity via interleukin-22. Immunity. (2013) 39:372–85. 10.1016/j.immuni.2013.08.003 23973224

[26] Parks OB Pociask DA Hodzic Z Kolls JK Good M. Interleukin-22 signaling in the regulation of intestinal health and disease. Front Cell Dev Biol. (2015) 3:85. 10.3389/fcell.2015.00085 26793707 PMC4710696

[27] Nolan-Kenney R Wu F Hu J Yang L Kelly D Li H. The association between smoking and gut microbiome in Bangladesh. Nicotine Tob Res. (2020) 22:1339–46. 10.1093/ntr/ntz220 31794002 PMC7364824

[28] Prakash A Peters BA Cobbs E Beggs D Choi H Li H. Tobacco smoking and the fecal microbiome in a large, multi-ethnic cohort. Cancer Epidemiol Biomarkers Prev. (2021) 30:1328–35. 10.1158/1055-9965.EPI-20-1417 34020999 PMC8254769

[29] Wang H Zhao JX Hu N Ren J Du M Zhu MJ. Side-stream smoking reduces intestinal inflammation and increases expression of tight junction proteins. World J Gastroenterol. (2012) 18:2180–7. 10.3748/wjg.v18.i18.2180 22611310 PMC3351767

[30] Kobayashi T Fujiwara K. Identification of heavy smokers through their intestinal microbiota by data mining analysis. Biosci Microbiota Food Health. (2013) 32:77–80. 10.12938/bmfh.32.77 24936365 PMC4034320

[31] Benjamin JL Hedin CR Koutsoumpas A Ng SC McCarthy NE Prescott NJ. Smokers with active Crohn's disease have a clinically relevant dysbiosis of the gastrointestinal microbiota. Inflamm Bowel Dis. (2012) 18:1092–100. 10.1002/ibd.21864 22102318

[32] Lee JH Lee J. Indole as an intercellular signal in microbial communities. FEMS Microbiol Rev. (2010) 34:426–44. 10.1111/j.1574-6976.2009.00204.x 20070374

[33] Gao J Xu K Liu H Liu G Bai M Peng C. Impact of the gut microbiota on intestinal immunity mediated by tryptophan metabolism. Front Cell Infect Microbiol. (2018) 8:13. 10.3389/fcimb.2018.00013 29468141 PMC5808205

[34] Satsangi J Silverberg MS Vermeire S Colombel JF. The Montreal classification of inflammatory bowel disease: controversies, consensus, and implications. Gut. (2006) 55:749–53. 10.1136/gut.2005.082909 16698746 PMC1856208

[35] Feuerstein JD Moss AC Farraye FA. Ulcerative Colitis. Mayo Clin Proc. (2019) 94:1357–73. 10.1016/j.mayocp.2019.01.018 31272578

[36] Magoc T Salzberg SL. FLASH: fast length adjustment of short reads to improve genome assemblies. Bioinformatics. (2011) 27:2957–63. 10.1093/bioinformatics/btr507 21903629 PMC3198573

[37] Camacho C Coulouris G Avagyan V Ma N Papadopoulos J Bealer K. BLAST+: architecture and applications. BMC Bioinformatics. (2009) 10:421. 10.1186/1471-2105-10-421 20003500 PMC2803857

[38] Huson DH Mitra S Ruscheweyh HJ Weber N Schuster SC. Integrative analysis of environmental sequences using MEGAN4. Genome Res. (2011) 21:1552–60. 10.1101/gr.120618.111 21690186 PMC3166839

